# Rapid molecular detection of *Senecavirus A* based on reverse transcription loop-mediated isothermal amplification (RT-LAMP) and CRISPR/Cas12a

**DOI:** 10.3389/fbioe.2025.1451125

**Published:** 2025-04-04

**Authors:** Chenghui Jiang, Huibao Wang, Rongxia Guo, Rui Yang, Xiaoming Li, Ping Liu, Jing Wang, Jincai Yang, Yanyan Chang, Qiaoying Zeng

**Affiliations:** ^1^ College of Veterinary Medicine, Gansu Agricultural University, Lanzhou, Gansu, China; ^2^ China Agricultural Veterinary Biological Science and Technology Co., Ltd., Lanzhou, Gansu, China; ^3^ College of Environmental Engineering, Gansu Forestry Voctech University, Tianshui, Gansu, China

**Keywords:** *Senecavirus A*, RT-LAMP, CRISPR/Cas12a, nucleic acid detection, visualisation

## Abstract

**Introduction:**

*Senecavirus A* (SVA), an emerging vesicular pathogen, is responsible for porcine idiopathic vesicular disease (PIVD). This disease is closely associated with porcine vesicular disease and acute neonatal piglet mortality, presenting a substantial threat to the global swine industry. At present, the absence of effective drugs or vaccines for treating the disease makes accurate diagnosis of SVA of paramount importance for the effective prevention and control of the disease.

**Methods:**

In this study, we combined reverse transcription loop-mediated isothermal amplification (RT-LAMP) and Clustered Regularly Interspaced Short Palindromic Repeats and CRISPR-associated protein12a (CRISPR/Cas12a) using a dual-labelled fluorescence quencher or fluorescent biotin single-stranded DNA reporter molecule to develop two rapid, reliable, and portable visual SVA assays: RT-LAMP-Cas12a-FQ and RT-LAMP-Cas12a-FB.

**Results:**

The two methods exhibited comparable detection limits, with 9.6 copies/μL achieved in 40 and 45 minutes, respectively. They did not cross-react with non-target nucleic acids extracted from other related viruses and showed high specificity for SVA RNA detection. Furthermore, the methods demonstrated satisfactory performance in detecting 69 porcine adventitious samples, with no significant differences from that of quantitative reverse transcription polymerase chain reaction (RT-qPCR).

**Discussion:**

In summary, the RT-LAMP-Cas12a-FQ and RT-LAMP-Cas12a-FB methods developed are promising for early detection and routine surveillance of porcine SVA in resource-poor areas.

## 1 Introduction

*Senecavirus A* (SVA), previously designated as the Seneca Valley virus, is a single-stranded RNA virus belonging to the genus *Senecavirus*, in the family *Picornaviridae*. Its viral particles exhibit icosahedral symmetry, are membraneless and have a diameter of approximately 22–27 nm (Segalés et al., 2017). The genome of SVA consists of approximately 7,300 nucleotides, including a 5′ non-coding region (5′-UTR) of 666 nucleotides, a 3′ non-coding region (3′-UTR) of 71 nucleotides, a unique open reading frame (ORF), and 3′ poly (A) tails (Ling et al., 2025). This virus was initially isolated from transformed foetal retinoblast (PER.C6) in 2002 (Hales et al., 2008). It was used in early antitumour therapy studies because of its ability to specifically target neurosecretory cells and its tumour-lytic effect (Reddy et al., 2007). In 2007 and 2012, SVA was associated with porcine vesicular disease and detected in pigs in Canada (Leme et al., 2017) and the United States (Guo et al., 2016), respectively. Since then, SVA outbreaks have been reported in several countries, including Vietnam (Arzt et al., 2019), Colombia (Sun et al., 2017), Brazil (Vannucci et al., 2015), Thailand (Saeng-Chuto et al., 2018), and China (Wu et al., 2017), critically threatening the substantial development of the global pig industry.

SVA is the causative agent of porcine idiopathic vesicular disease (PIVD). The virus can infect pigs of different ages, and the typical symptoms of infection include severe blisters and ulcers on the skin, nose, oral mucosa, hooves, and coronary band and acute death in newborn piglets (Liu et al., 2020). The clinical symptoms are similar to those of foot-and-mouth disease, vesicular stomatitis, vesicular exanthema, and swine vesicular disease and are usually accompanied by mixed infections, complicating clinical differential diagnosis (Zhang et al., 2018).

The rapid and accurate detection of SVA is crucial to preventing and controlling its associated disease. Polymerase chain reaction (PCR), real-time reverse transcription PCR (RT-PCR) (Zhang et al., 2019; Fowler et al., 2017), quantitative reverse transcription PCR (RT-qPCR) (Bracht et al., 2016; Dall Agnol et al., 2017), and enzyme-linked immunosorbent assay (ELISA) (Dvorak et al., 2017) are among the contemporary traditional techniques that are frequently employed for SVA detection. Unfortunately, these techniques have drawbacks that make them very difficult to use in clinical settings, including the need for sophisticated devices, time-consuming procedures, and trained personnel (Table 1). Therefore, developing a convenient, highly sensitive, specific, and reliable SVA detection method for effective surveillance of viruses and epidemiological investigations is crucial, particularly in areas with limited resources at the grassroots level.

**TABLE 1:** Different assays to detect SVA.

Methods	Targets	Detection limits	Detection time	Bulky instrumentation required	Refs.
RT-PCR	3D	0.79 TCID50/ml	>1 h	Yes	Zhang et al. (2019)
RT-qPCR	VP1	1.3 × 10^1^ copies/μL	>1 h	Yes	Bracht et al. (2016)
Digital PCR	3D	1.53 ± 0.22 copies/reaction	>2 h	Yes	Pinheiro-de-Oliveira et al. (2019)
Nested-PCR	VP1	0.0181 ng/μL	>1 h	Yes	Feronato et al. (2018)
ELISA	VP2	10 pg–1000 pg/mL	>2 h	Yes	Dvorak et al. (2017)
RT-LAMP-Cas12a-FQ	5′-UTR	9.6 × 10^0^ copies/μL	40 min	No	This study
RT-LAMP-Cas12a-FB	5′-UTR	9.6 × 10^0^ copies/μL	45 min	No	This study

Loop-mediated Isothermal Amplification (LAMP) is a technique that involves using a strand-substituted polymerase and four to six pairs of specific primers for target genes (DNA or RNA) to achieve rapid amplification only under constant temperature conditions (approximately 60°C–65°C) (Gieroń et al., 2023). This technique does not require specialised laboratory equipment, and combined with its high sensitivity, specificity, and straightforward operation, it has caused a growing interest in its potential applications (Soroka et al., 2021; Notomi et al., 2000). Reverse transcription LAMP (RT-LAMP), which combines LAMP with reverse transcription for direct RNA detection, represents the current direction for the development of this technology (Storms et al., 2023). However, cross-contamination is a significant limitation to its application (Wang et al., 2024).

The CRISPR-Cas system is composed of clustered regularly interspaced short palindromic repeats (CRISPR)-associated protein (Cas), an acquired immunity system found in bacteria and archaea. This system functions through CRISPR RNA (crRNA)-guided nucleases to confer sequence-specific resistance against foreign genetic elements (Amitai and Sorek, 2016). It is generally divided into two types according to the structural and functional attributes of Cas proteins. Class I Cas proteins are effector complexes composed of multiple subunits (Mao X. et al., 2022), and class II Cas proteins are single effector proteins including Cas9, Cas12a, Cas13a, and Cas14 systems (Makarova et al., 2020; Gootenberg et al., 2018).

The simplicity of the effector structure of the Class II CRISPR-Cas system has enabled it to show greater potential for application in the fields of genome editing, diagnosis, and therapy (Sahel et al., 2024; Zhang et al., 2024). In this class, Cas9, the most extensively employed gene-editing enzyme, relies on the assembly of single guide RNAs (sgRNAs) through the combination of crRNAs and trans-activating CRISPR RNAs (tracrRNAs) (Cong et al., 2013). These sgRNAs direct Cas9 to recognise 5′-NGG protospacer-adjacent motifs (PAM) on target DNA sequences, thereby activating Cas9’s capacity to cleave double-stranded DNA (dsDNA). Cas12a requires only single-stranded crRNA and specifically recognises 5′-TTTN PAM sequences on DNA (e.g., LbCas12a) to achieve targeted cleavage while exhibiting non-specific cleavage activity (trans-cleavage activity) on nearby single-stranded DNA (ssDNA) (Zetsche et al., 2015). In contrast to Cas12a, Cas13a is an RNA-mediated RNA endonuclease comprising two HEPN structural domains that specifically recognise and cleave target RNA under the guidance of crRNA, and is capable of indiscriminately cleaving nearby single-stranded RNA (ssRNA) (Zhong et al., 2025; Li et al., 2025). The Cas14 represents a different type of CRISPR RNA effector that functions by targeting ssDNA. It does not require a PAM and is suitable for low abundance DNA detection (Zhao et al., 2024). Among these Cas enzymes, Cas12a and Cas13a have been identified by researchers as the most promising candidates for utilisation in diagnostic techniques due to their unique trans-cutting activity, with Cas12a in particular being superior in terms of sensitivity and stability (Talwar et al., 2021; Xie et al., 2021; Chen et al., 2022).

In CRISPR/Cas12a system, PAM are recognised by the Cas12a nuclease, which is guided by crRNA to cleave dsDNA at the target site with preferentiality (Paul and Montoya, 2020; Fan et al., 2023). In this instance, the adjacent ssDNA that has been labelled with biotin or quenched fluorescence is indiscriminately cleaved by the activated Cas12a nuclease (Kim et al., 2021). The cleavage results can be visualized using a UV fluorescence reader or lateral flow test strips, facilitating the detection of target genes (Hu et al., 2024). Nevertheless, the CRISPR-Cas12a system is not sufficiently sensitive to detect low concentrations of nucleic acids (Zhou et al., 2023). Consequently, it is frequently combined with isothermal amplification techniques, such as LAMP, for enhanced sensitivity (Lei et al., 2022; Misra et al., 2022). The combination of LAMP and CRISPR/Cas12a overcomes the limitations of using these technologies alone for nucleic acid detection, demonstrating optimal complementarity (Mao et al., 2024). The integration of LAMP amplification into the CRISPR/Cas12a system has led to an improvement in the sensitivity of the detection process. Conversely, the CRISPR/Cas12a system effectively eliminated the non-specific amplification signal of LAMP (Yang et al., 2022). To date, a novel assay combining LAMP and CRISPR/Cas12a has been successfully applied in several fields, including the detection of bacterial (Mao Z. et al., 2022; Shin et al., 2024), parasitic (Wei et al., 2023; Li et al., 2022), viral (Broughton et al., 2020; Wang et al., 2021; Yuan et al., 2020), and genetically modified products (Wang et al., 2022; Wu et al., 2020). This method has great potential for developmental applications.

In this study, we report two rapid and portable visualisation assays based on the advantages of RT-LAMP and CRISPR/Cas12a: RT-LAMP-Cas12a-FQ and RT-LAMP-Cas12a-FB. Following sample processing, the assay results were obtained using UV irradiation or lateral flow test strips within 45 min. These methods can be a promising alternative assay for SVA and provide valuable tools for SVA monitoring and control.

## 2 Materials and methods

### 2.1 Viruses and clinical samples

SVA FJ, foot-and-mouth disease virus (FMDV) O/MYA98, classical swine fever virus (CSFV), porcine epidemic diarrhea virus (PEDV) CV777, porcine circovirus 2 (PCV2) LG, and porcine reproductive and respiratory syndrome (PRRSV) JXA1 vaccine strains were maintained at the State Key Laboratory of the Lanzhou Veterinary Research Institute of Agricultural Sciences, China. A total of 69 blood and blister fluid samples were collected from three farms in Gansu, where pigs exhibited symptoms of diarrhoea and blistering. The clinical samples were stored at a temperature of −80°C in order to facilitate the subsequent nucleic acid extraction process.

### 2.2 Preparation of RT-LAMP primers and crRNAs

SVA gene sequences were obtained from the National Centre for Biotechnology Information (NCBI) database, while the 5′ untranslated regions (5′-UTR) sequences were subjected to Basic Local Alignment Search Tool analysis. The SVA genome accession numbers are MN615881.1, MN423334.1, MN171528.1, MN812960.1, MN433300.1, MK333636.1, MN233020.1, MH634532.1, MH634511.1, MK802892.1, MK039162.1, MF189001.1, MG983756.1, MG765558.1, MF615510.1, OR759780.1, OR703632.1, and MF615503.1. Primers for RT-LAMP were designed based on the 5′-UTR conserved region of the SVA genome using the online software Primer Explorer V 5.0 (http://primerexplorer.jp/). Three crRNAs were designed in the 5′-UTR conserved region of the SVA using the online tool CRISPOR (http://crispr.tefor.net/). The crRNAs were designed with the LbaCas12a scaffolding sequence (UAA​UUU​UCU​ACU​AAG​UGU​AGA​U) appended to the 5′ end of each crRNA. The RNA standard amplification primers ST-RNA-F and ST-RNA-R were designed by adding the T7 promoter sequence (TAA​TAC​GAC​TCA​CTA​TAG​GG) to the 5′ end of the primers. All primer sequences were synthesised by Jin Wei Zhi Biological Science and Technology Co., Ltd. (Tianjin, China) and are listed in Table 2.

**TABLE 2:** Sequence information of primers and crRNAs.

Primer name	Sequences(5′-3′)	Amplification length
F3	TTA​GCG​GGT​CTC​CTC​ACA​A	214 bp
B3	GCTGATCTGTGCTGCTGG	
FIP	CGCCGTTTGCGTTATGACTC GACTTGAACCCTCTTGGCTACC	
BIP	GGCAACACTGCCATAAACACG CCGGAGGGTCAGATTTGGTC	
LF	GCA​GAG​TTT​TCC​ATT​TCT​TCG​GTA​T	
LR	AAT​CAT​CAC​TGG​GTG​TGT​TGT​G	
ST-RNA-F	TAATACGACTCACTATAGGG TTAGCGGGTCTCCTCACAA	214 bp
ST-RNA-R	GCTGATCTGTGCTGCTGG	
crRNA1	UAA​UUU​CUA​CUA​AGU​GUA​GAU​CGU​UAU​GAC​UCG​AUC​AGC​AGA​GU	
crRNA2	UAA​UUU​CUA​CUA​AGU​GUA​GAU​UCA​CCU​UAG​AAC​UUG​GGA​GAA​CC	
crRNA3	UAA​UUU​CUA​CUA​AGU​GUA​GAU​UGA​GGA​GAC​CCG​CUA​AUC​CGC​CC	
ssDNA FQ reporter	5′-FAM-TTATT-BHQI-3′ (for RT-LAMP-Cas12a-FQ assay)	
ssDNA FB reporter	5′-FAM-TTTTTTTATTTTTTT-Biotin-3′ (for RT-LAMP-Cas12a-FB assay)	

### 2.3 Preparation of recombinant plasmid and standard RNA

RNA was extracted from the SVA using a Viral RNA Extraction Kit (OMEGA, United States) in accordance with the manufacturer’s instructions. Subsequently, the SVA target fragment was amplified according to the instructions provided in the RT-PCR kit (TaKaRa, Japan) (Supplementary Figure S1A). This was ligated into the plasmid pMD19-T using T4 ligase (TaKaRa, Japan). The recombinant plasmid (pMD19-JC) was sequenced and was found to contain the target fragment without mutations.

PCR amplification was performed using ST-RNA-F and ST-RNA-R as primers (Table 2) and linearised pMD19-JC as a template. The amplified products were identified using 0.9% agarose gel electrophoresis and subjected to gel recovery using a gel recovery kit (TianGen, Beijing, China). The recovered products were used for T7 transcription at 37°C for 5 h. The resulting transcription products were digested with DNase I (Solarbio, Beijing, China) and purified using an RNA Purification and Recovery Kit (SHENGONG, Shanghai, China) to obtain standard RNA (ST-RNA). The nucleic acids of all viruses were stored at −80°C for further analyses.

### 2.4 RT-LAMP assay

The RT-LAMP reactions were conducted following the instructions in the RT-LAMP kit (HaiGene, Harbin, China). The reaction mixture, comprising 1× ThermoPol buffer, 7 μM MgSO^4^, 8 U Bst 4.0 DNA/RNA Polymerase, 1.4 μM dNTP mixture, and primer mixture (1.6 μM FIP and BIP, 0.2 μM F3 and B3, and 0.8 μM Loop F and Loop R), was prepared in a volume of 25 μL. Additionally, 2.5 μL of RNase-free ddH_2_O and 0.9 μL of template DNA/RNA (RT-LAMP reaction if RNA, LAMP reaction if DNA) were added. A screening process was used to identify the optimal conditions for RT-LAMP. The results demonstrated that the optimal parameters for the Mg^2+^ concentration, annealing temperature, and reaction time were 7 mM, 65°C, and 30 min, respectively (Supplementary Figures S1B–D, respectively).

### 2.5 RT-LAMP-Cas12a-FQ assay

The 20 μL RT-LAMP-Cas12a-FQ assay detection reaction mix included 1× HOLMES Buffer, 250 nM LbCas12a (TOLOBIO, Shanghai, China), 250 nM crRNA, 250 nM ssDNA FQ reporter, 16.5 μL RNase-free ddH_2_O, and 0.25 μL RT-LAMP amplification product. The reaction tubes were incubated at 37°C for a period of 15–30 min. The RT-LAMP-Cas12a-FQ assay was analysed using a gel imaging system with a UV channel. Fluorescence intensity was quantified using ImageJ software. Among the three designed crRNAs, one with high activity was identified. The optimal concentrations of crRNA, ssDNA FQ reporter, and LbCas12a, reaction time, and reaction temperature for the Cas12a cleavage reaction were determined using the RT-LAMP-Cas12a-FQ method.

### 2.6 RT-LAMP-Cas12a-FB assay

The ssDNA FB reporter was diluted into five concentrations (5 nM, 50 nM, 500 nM, 5 μM, and 50 μM) following the lateral flow chromatography test strip instructions (Tiosbio, Beijing, China). Subsequently, each dilution reaction tube was inserted into test strips. The concentration of the ssDNA FB reporter that resulted in only the C line showing colour and not the T line was used as the negative control concentration.

The RT-LAMP-Cas12a-FB reactions were performed using optimised concentrations of the ssDNA FB reporter. The reaction mixture comprised 1× HOLMES Buffer, 200 nM LbCas12a (TOLOBIO, Shanghai, China), 100 nM crRNA, 50 nM ssDNA FB reporter, 17.25 μL RNase-free ddH_2_O, and 0.25 μL LAMP or RT-LAMP amplification product, with a volume of 20 μL. Test paper strips were inserted into each group of tubes at 5, 10, 15, 20, 25, 30, and 35 min after the reaction. Changes in the test strips were observed after 5 min to determine the optimal time for the RT-LAMP-Cas12a-FB assay.

The criteria for evaluating the test strip results were as follows: if only the C line appeared, it indicated a negative result, and the reaction product did not contain the nucleic acid of the tested virus; if the C and T lines were present, the result was positive, and the reaction product contained the tested viral nucleic acid; if only the T line were present, this also indicated a positive result and a high concentration of viral nucleic acid was detected in the reaction product.

### 2.7 RT-qPCR assay

RNA samples were amplified using a LightCycler96 real-time quantitative PCR instrument (Roche, United States) with primers F3 (5′-TTA​GCG​GTC​TCC​TCA​CAA-3′) and B3 (5′-GCT​GAT​CTG​TGC​TGC​TGG​G-3′) to enable comparison with our established standard method (Supplementary Figure S2A). The RT-qPCR reaction mixture (20 µL) consisted of 10 µL of 2× One Step TB Green Buffer (TaKaRa, Japan), 0.8 µL of PrimerScript 1 Step Enzyme Mix 2, 0.8 µL of F3 Primer and B3 Primer (10 µM), 0.4 µL of ROX Reference Dye or Dye II (50×), 2.2 µL RNA template and 5.0 µL RNase-free ddH_2_O. The amplification conditions were as follows: 42°C for 6 min and 94°C for 15 s, followed by 43 cycles of 94°C for 5 s and 62°C for 40 s.

### 2.8 Sensitivity and specificity assays

The sensitivities of RT-LAMP-Cas12a-FQ and RT-LAMP-Cas12a-FB were evaluated using pMD19-JC and ST-RNA as templates, respectively. The plasmid concentration was converted to copy number, diluted in a 10-fold gradient, and 8.3 × 10^8^ to 8.3 × 10^−1^ copies/μL were used as templates for LAMP amplification. The reaction products were subsequently detected using LAMP-Cas12a-FQ and LAMP-Cas12a-FB. The ST-RNA concentration was converted to copy number, ranging from 9.6 × 10^6^ to 9.6 × 10^−1^ copies/μL. Subsequently, the amplification products were detected using the RT-LAMP-Cas12a-FQ and RT-LAMP-Cas12a-FB methods following the same steps. The qRT-PCR was performed in parallel.

Six nucleic acid samples from SVA, FMDV, CSFV, PCV2, PEDV, and PRRSV were employed to assess the specificity of the RT-LAMP-Cas12a-FQ and RT-LAMP-Cas12a-FB assays, with RNase-free ddH_2_O serving as the negative control.

### 2.9 Reproducibility evaluation of the SVA assay

The reproducibility of the RT-LAMP-Cas12a-FQ and RT-LAMP-Cas12a-FB methods was assessed by performing three intra-batch and three inter-batch reproducibility tests using ST-RNA at concentrations. The RT-LAMP-Cas12a-FQ and RT-LAMP-Cas12a-FB methods were tested at concentrations of 9.6 × 10^6^, 9.6 × 10^4^, and 9.6 × 10^2^copies/μL, and sterile water was used as a negative control. The assay was repeated three times at different time points.

### 2.10 SVA clinical sample testing

Sixty-nine clinical samples were removed from a temperature of −80°C and left to dissolve and centrifuged briefly. Nucleic acids were extracted according to the instructions provided with the kit and the RT-LAMP reaction was performed. The RT-LAMP products were used as templates and detected using the RT-LAMP-Cas12a-FQ and RT-LAMP-Cas12a-FB methods established in this study, along with a parallel RT-qPCR assay.

### 2.11 Statistical analysis

The graphs were generated using GraphPad Prism 9.0 software. Statistical analyses were conducted using IBM SPSS software with a one-way ANOVA. The statistical significance of the results was determined by comparing the experimental and control groups. The following statistical significance levels were used: Data are presented as mean ± SEM (n = 3), ****P < 0.0001, ***P < 0.001, **P < 0.01, and *P < 0.05. The designation “ns” indicates no statistically significant difference.

## 3 Results

### 3.1 Principle of CRISPR/Cas12a-based detection of SVA

**FIGURE 1:**
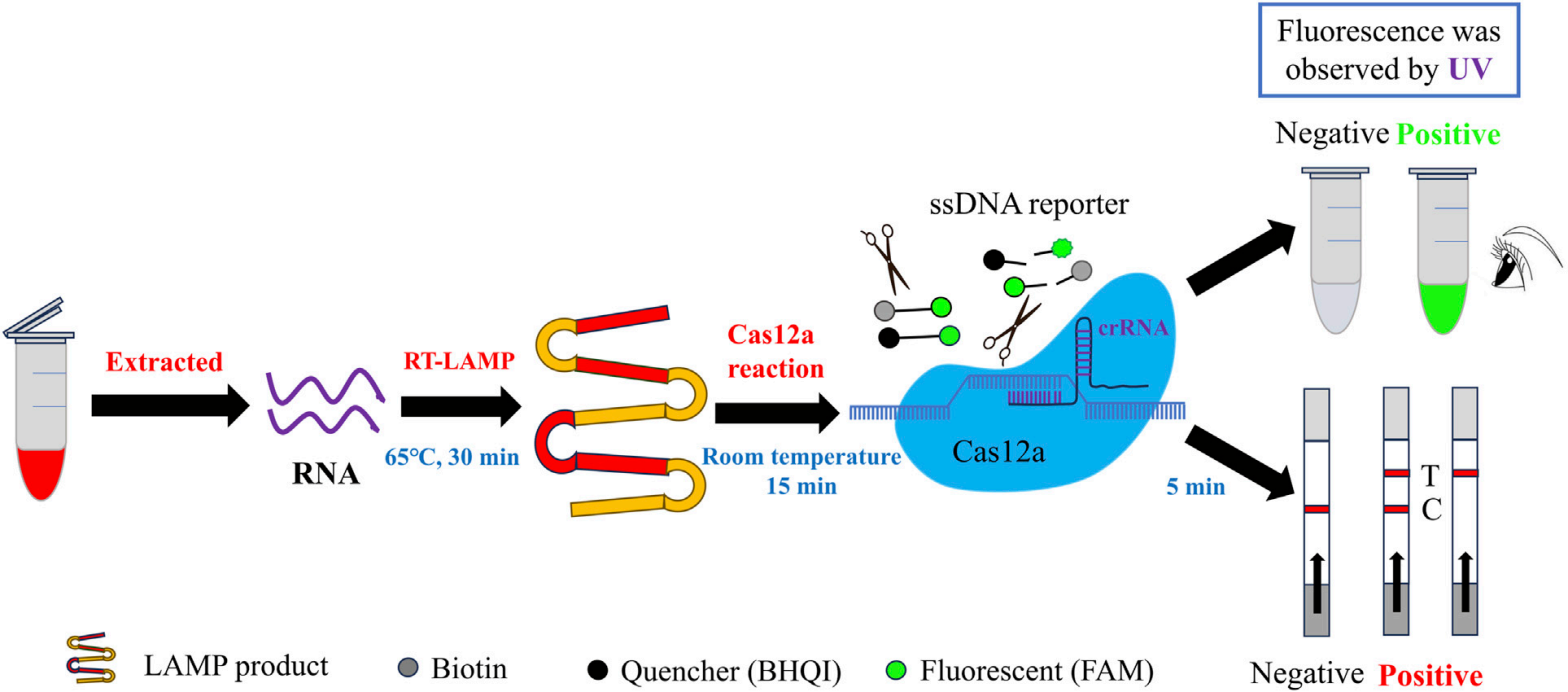
Schematic of RT-LAMP-Cas12a-FQ and RT-LAMP-Cas12a-FB assays for SVA.

The fundamental principle underlying the CRISPR/Cas12a-based detection of SVA is illustrated in Figure 1. The nucleic acid RNA extracted from the samples was subjected to a RT-LAMP reaction, which generated double-stranded DNA templates for subsequent CRISPR/Cas12a reactions. Once the CRISPR/Cas12a system is operational, the crRNA initially guides the Cas12a protein to recognise the target dsDNA, which then activates and binds to the Cas12a protein to form a ternary complex (Selvam et al., 2021). Subsequently, the Cas12a protein proceeds to cleave the target dsDNA, while its trans-cutting activity is likewise activated, leading to the cleavage of nearby non-target ssDNA (Li et al., 2018). If the ssDNA FQ reporter is modified at both ends to add a fluorescent moiety (5′-FAM) and a quenching moiety (3′-BHQI), the reporter remains intact. In this configuration, the FAM is in close proximity to the BHQI, which suppresses its fluorescence primarily through energy transfer of the Förster type (Hu et al., 2024). Once Cas12a has initiated its cross-cutting activity and is functioning, the FAM separates from the BHQI, thereby producing a fluorescent signal (Mukama et al., 2020). This allows for observation of the fluorescent signal resulting from UV irradiation. If a 5′-FAM and a biotin molecule (3′-Biotin) are appended to the extremities of the ssDNA FB reporter, the activated Cas12a protein will cleave the ssDNA reporter gene laterally in a similar manner, allowing the FAM to be separated from the biotin (Lee and Oh, 2023). This enables the presentation of the cleavage results by lateral flow test strips. This strategy enabled the development of two CRISPR-Cas12a methods for the detection of SVA: RT-LAMP-Cas12a-FQ and RT-LAMP-Cas12a-FB.

### 3.2 Identification of conserved and highly active crRNA for SVA detection

**FIGURE 2:**
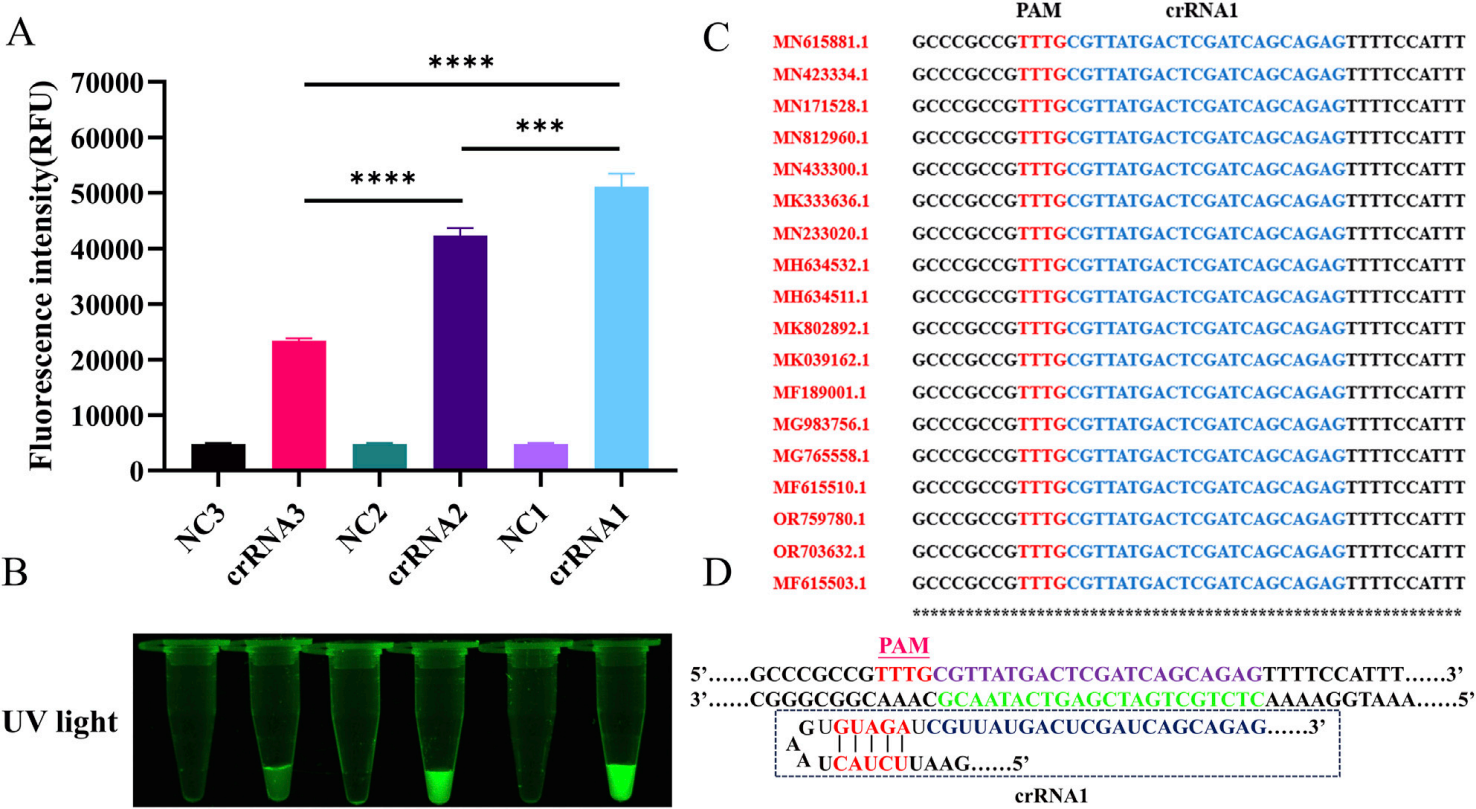
The screening of highly active crRNA by RT-LAMP-Cas12a-FQ method. **(A)** Histogram of fluorescence intensity values of the CRISPR/Cas12a reaction for crRNAs screening (Fluorescence intensity values were calculated using ImageJ software). **(B)** Visual fluorescence results of the CRISPR/Cas12a reaction for crRNAs screening. **(C)** Analysis of conserved sequences of crRNA1 different SVA strains (only 18 strains are shown here). **(D)** Schematic diagram of crRNA1 target sequence design.

Two novel approaches for detecting SVA were developed based on the RT-LAMP technology and CRISPR/Cas12a reporter systems: fluorescence (RT-LAMP-Cas12a-FQ) and lateral flow test strip (RT-LAMP-Cas12a-FB) methods (Figure 1). The highly active crRNAs were screened using the RT-LAMP-Cas12a-FQ system (Figure 2). After 30 min of reaction at 37°C, the results were observed under UV light. Tubes containing the three crRNA systems emitted green fluorescence, indicating targeting activity of the three crRNAs (Figure 2A). Fluorescence intensity measurements revealed that crRNA1 exhibited the highest targeting activity, followed by crRNA2 and crRNA3 (Figure 2B). Furthermore, the crRNA1 sequence was highly conserved among the sequences of different SVA strains published in the NCBI database (Figure 2C). Therefore, high-efficiency crRNA1 was selected for subsequent experiments.

### 3.3 Optimisation of the CRISPR/Cas12a cleavage assay

**FIGURE 3:**
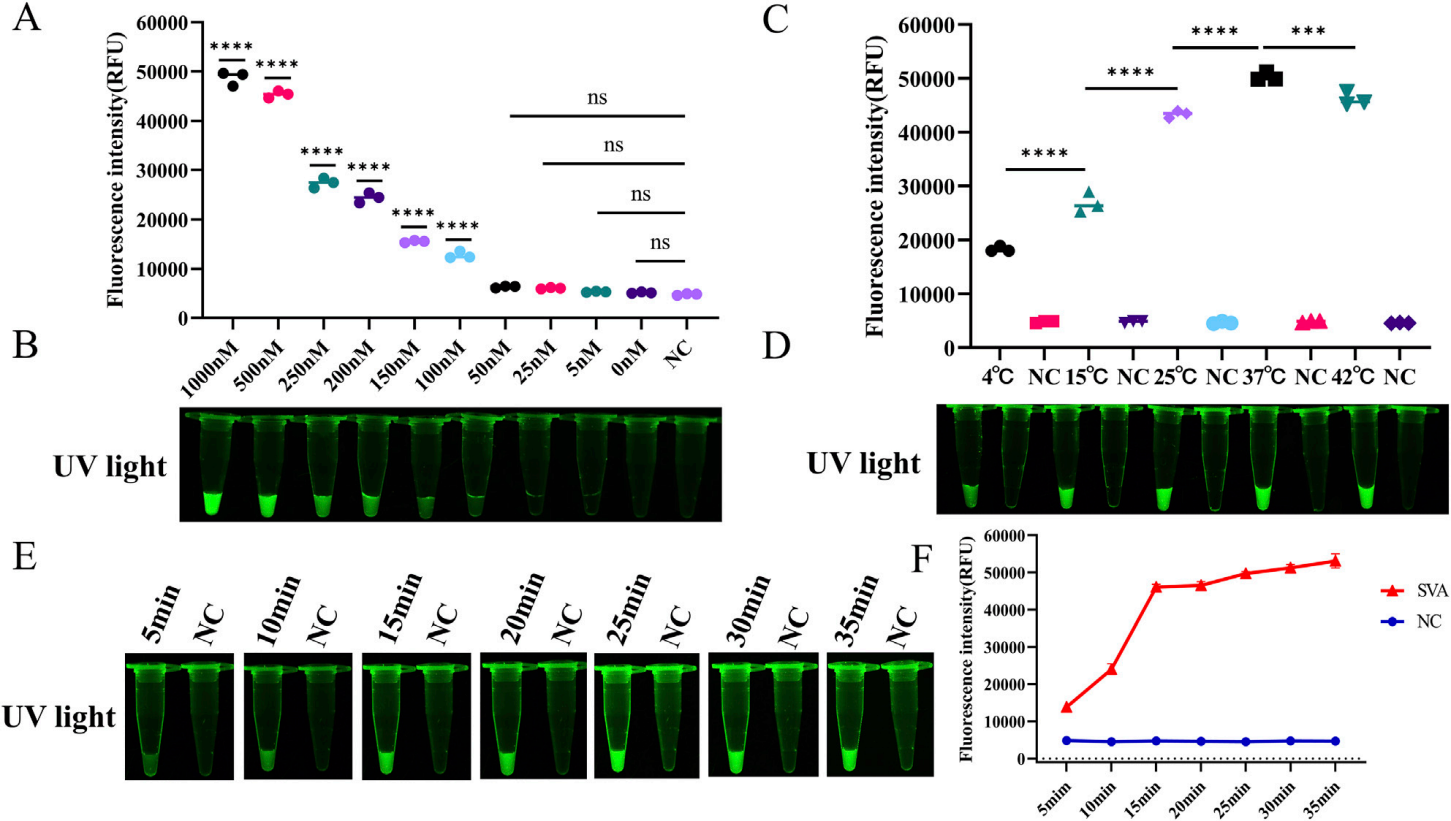
Optimisation of reaction conditions for the RT-LAMP-Cas12a-FQ method. **(A)** Histogram of fluorescence intensity values of the CRISPR/Cas12a reaction at varying concentrations of ssDNA FQ reporter (Fluorescence intensity values were calculated using ImageJ software). **(B)** Visual fluorescence outcomes of the CRISPR/Cas12a reaction at varying ssDNA FQ reporter concentrations. **(C)** Histogram of fluorescence intensity values of the CRISPR/Cas12a reaction at varying temperatures. **(D)** Visual fluorescence results of the CRISPR/Cas12a reaction at varying temperatures. **(E)** Visual fluorescence outcomes of the CRISPR/Cas12a reaction times. **(F)** Line graphs of the fluorescence intensity values of the CRISPR/Cas12a reaction times.

The assay conditions for RT-LAMP-Cas12a-FQ were optimised. The fluorescence values gradually increased as the concentration of the ssDNA FQ reporter increased (Figures 3A, B). To maximise the fluorescence release and reduce costs, we used an ssDNA FQ reporter concentration of 500 nM in subsequent experiments. Orthogonal tests were conducted using LbCas12a concentrations ranging from 25 to 250 nM and crRNA concentrations ranging from 25 to 250 nM. The fluorescence intensity was the highest at a LbCas12a concentration of 200 nM and a crRNA concentration of 100 nM (Supplementary Figure S3). The CRISPR-Cas12a reactions were performed at temperatures ranging from 4°C to 42°C. Green fluorescence was produced in the reaction tubes at different temperatures; however, the fluorescence intensity was highest at 37°C (Figures 3C, D). Therefore, 37°C is the optimal temperature for the RT-LAMP-Cas12a-FQ method. However, maintaining a constant temperature of 37°C can be challenging and cumbersome. All experiments in this study were conducted at room temperature (10°C–30°C) for convenience. Furthermore, the reaction time of the Cas12a-mediated cleavage experiment was optimised. The fluorescence intensity of the CRISPR-Cas12a reaction was measured at 5, 10, 15, 20, 25, 30 and 35 min. The results demonstrated that the fluorescence intensity reached a stable peak after 15 min, thereby substantiating the assertion that the optimal detection time for the RT-LAMP-Cas12a-FQ method is 15 min (Figures 3E, F).

**FIGURE 4:**
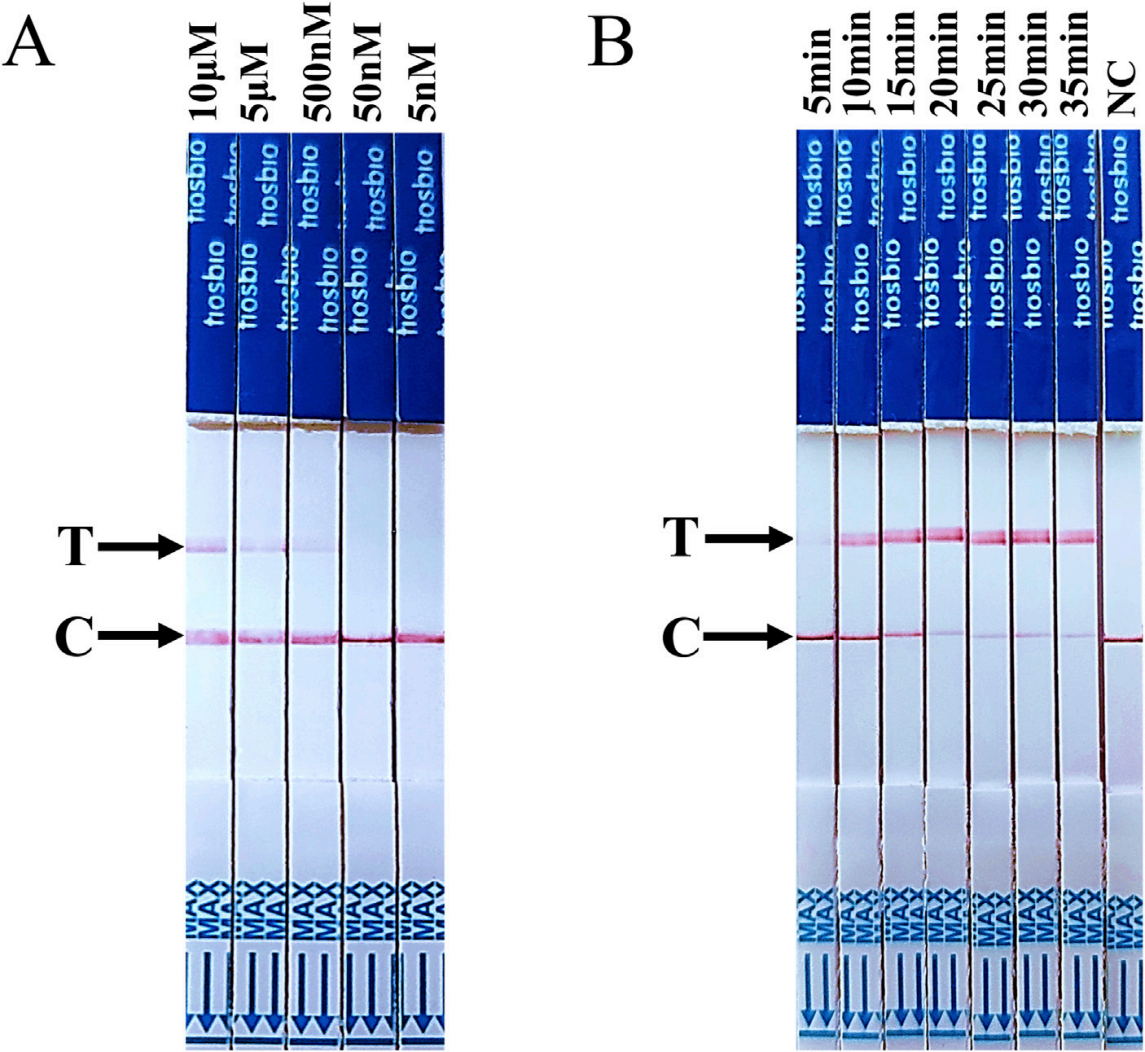
Optimisation of ssDNA FB reporter concentration and CRISPR/Cas12a reaction time for the RT-LAMP-Cas12a-FB method. **(A)** The findings on changes in lateral flow strips at different concentrations of ssDNA FQ reporters. **(B)** The findings on changes in lateral flow strips at different times of the CRISPR/Cas12a reaction. “C” indicates control line, “T” indicates test line.

Subsequently, the assay conditions for RT-LAMP-Cas12a-FB were optimised. To prevent false-positive results, conducting a screening process to determine the optimal concentration of ssDNA FB reporter for the RT-LAMP-Cas12a-FB assay is essential. The T line of the test strip did not appear when the ssDNA FB reporter concentration was less than 50 nM (Figure 4A). This indicates that 50 nM was the optimal concentration of ssDNA FB reporter for the RT-LAMP-Cas12a-FB assay. Test strips were inserted into each group of tubes after 5, 10, 15, 20, 25, 30, and 35 min of the CRISPR/Cas12a reaction. At 15 min of the CRISPR/Cas12a reaction, the T line of the test paper was discernible (Figure 4B), suggesting that 15 min was the optimal duration for the RT-LAMP-Cas12a-FB method of the Cas12a reaction.

### 3.4 Sensitivity evaluation of the SVA assay

The sensitivity of the SVA assay was evaluated using DNA as a template. The pMD19-JC was diluted from 8.3 × 10^8^ to 8.3 × 10^−1^ copies/µL for LAMP amplification. The results showed that both the LAMP-Cas12a-FQ and LAMP-Cas12a-FB methods, which are based on LAMP combined with CRISPR-Cas12a, had a lowest detection limit of 8.3 copies/µL when pMD19-JC was used as a template (Supplementary Figures S4A–C). In the LAMP-Cas12a-FQ method, the log_10_ (copies/µL) of pMD19-JC demonstrated a linear correlation with the fluorescence intensity within the range of 8.3 × 10^2^ to 10^6^ (y = 8,194x + 10,062, R^2^ = 0.9614), where y represents the fluorescence intensity and x represents the log_10_ (copies/µL) (Supplementary Figure S5A).

**FIGURE 5:**
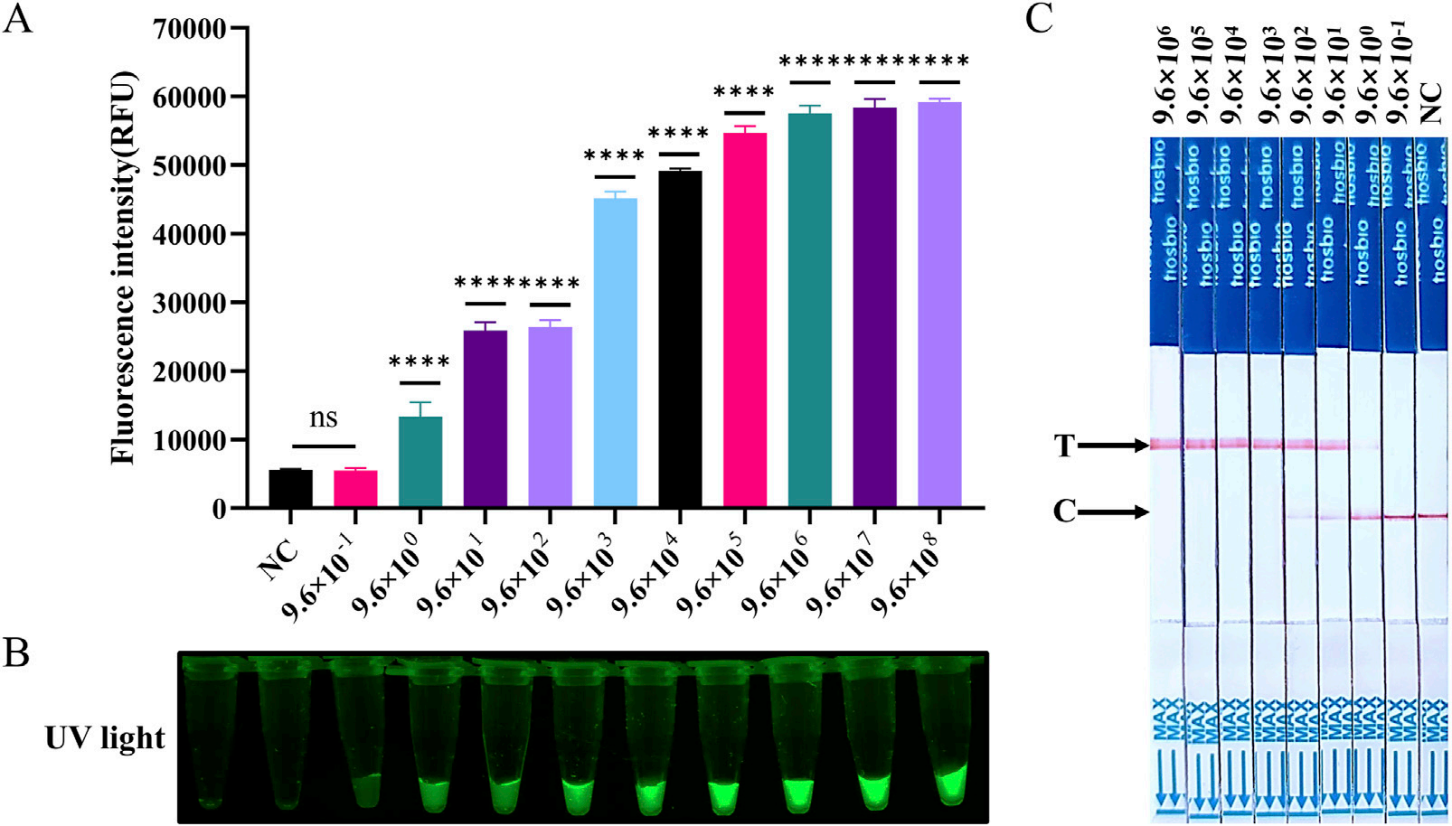
Evaluation of the sensitivity of RT-LAMP-Cas12a to detect SVA. **(A)** Histogram of fluorescence intensity values of the sensitivity of the RT-LAMP-Cas12a-FQ method (ST-RNA gradient ranging from 9.6 × 10^8^ to 9.6 × 10^−1^ copies). **(B)** Visual fluorescence outcomes of the sensitivity of the RT-LAMP-Cas12a-FQ method (ST-RNA gradient ranging from 9.6 × 10^8^ to 9.6 × 10^−1^ copies). **(C)** Sensitivity of the RT-LAMP-Cas12a-FB method (ST-RNA gradient ranging from 9.6 × 10^6^ to 9.6 × 10^−1^ copies).

The sensitivity of the SVA assay was further evaluated using RNA as a template. The ST-RNA was employed as a template for RT-LAMP amplification following a tenfold dilution from 9.6 × 10^6^ to 9.6 × 10^−1^ copies/µL. The results indicated that the lowest detection limits of RT-LAMP-Cas12a-FQ and RT-LAMP-Cas12a-FB for SVA were consistent with those of RT-qPCR methods. The sensitivity of all three methods was found to be 9.6 copies/µL (Figures 5A–C; Supplementary Figure S2B). In the RT-LAMP-Cas12a-FQ method, the log_10_ (copies/µL) of ST-RNA was found to be linearly correlated with the fluorescence intensity in the range of 9.6 × 10^2^ to 10^6^ (y = 7,854x + 7,321, R^2^ = 0.9612, where y is fluorescence intensity and x is log_10_ (copies/µL)) (Supplementary Figure S5B).

### 3.5 Specificity evaluation of the SVA assay

**FIGURE 6:**
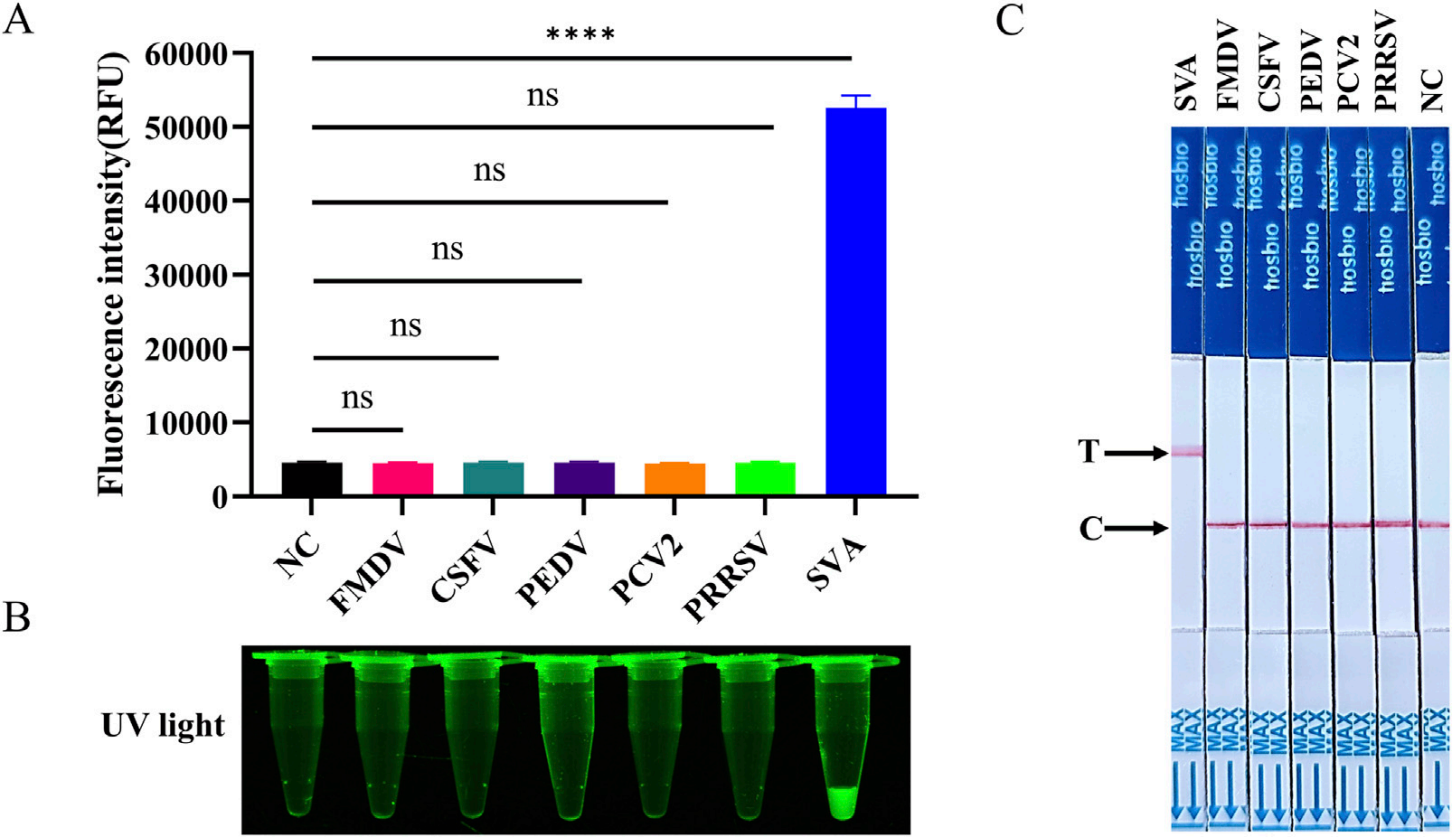
Evaluation of the specificity of RT-LAMP-Cas12a to detect SVA. **(A)** Histogram of fluorescence intensity values of RT-LAMP-Cas12a-FQ method to SVA detection specificity. **(B)** Visual fluorescence outcomes of RT-LAMP-Cas12a-FQ method to SVA detection specificity. **(C)** Specificity of RT-LAMP-Cas12a-FB method to detect SVA.

To ascertain the specificity of the RT-LAMP-Cas12a-FQ and RT-LAMP-Cas12a-FB methods established in this study, nucleic acids were extracted and tested from SVA, FMDV, CSFV, PEDV, PCV2, and PRRSV samples (PCV2 is a DNA virus and the rest are RNA viruses). The results demonstrated a positive outcome for SVA and negative results for the remaining viruses and control water samples (Figure 6). This indicates that the RT-LAMP-Cas12a-FQ and RT-LAMP-Cas12a-FB methods demonstrate high specificity for SVA detection.

### 3.6 Reproducibility evaluation of the SVA assay

The reproducibility results demonstrated that the RT-LAMP-Cas12a-FQ method and RT-LAMP-Cas12a-FB method exhibited the capacity to reliably detect positive signals, at concentrations of 9.6 × 10^6^, 9.6 × 10^4^, and 9.6 × 10^2^ copies/μL.The RT-LAMP-Cas12a-FQ method demonstrated coefficient of variation (CV) within batch and between batch of less than 5% and 10%, respectively (Supplementary Table S1). The collective analysis of these results indicates that the reproducibility of the established methods is satisfactory.

### 3.7 Detection of SVA in clinical samples

To assess the performance of the RT-LAMP-Cas12 assay, 69 blood and blister fluid samples were collected from pigs exhibiting symptoms of the disease from three farms in Gansu Province. RNA was extracted from the samples using an RNA/DNA extraction kit and detected using RT-LAMP-Cas12a-FQ and RT-LAMP-Cas12a-FB and RT-qPCR. The findings from the three methods were consistent, with 18 samples yielding positive results and 51 samples yielding negative results. The overall positive rate was 26.1% (Table 3). A comparison of the RT-LAMP-Cas12a-FQ and RT-LAMP-Cas12a-FB methods with RT-qPCR revealed that both had a sensitivity and specificity of 100%, with a compliance rate of up to 100% (Supplementary Table S2). These results demonstrated that the RT-LAMP-Cas12a-FQ and RT-LAMP-Cas12a-FB methods developed in this study can be applied in clinical settings.

**TABLE 3:** Test results of SVA clinical samples.

Fams	NO. of samples	Positive/Negative			Positive rate (%)	Coincidence rate (%)
		RT-qPCR	RT-LAMP-Cas12a-FQ	RT-LAMP-Cas12a-FB		
A	23	5/18	5/18	5/18	27.8	100
B	25	6/19	6/19	6/19	31.6	100
C	21	7/14	7/14	7/14	50	100
Total	69	18/69	18/69	18/69	26.1	100

## 4 Discussion

SVA is a single-stranded positive-sense RNA virus. Inter-viral recombination, which contributes to the genetic diversity of SVA, has a high mutation potential, making it challenging to predict the virus’s future epidemiology, pathogenicity, and risk of cross-species transmission (Zhang et al., 2018). More than half of China’s provinces, municipalities, and autonomous regions are affected by SVA infections (Burke, 2016; Piepenburg et al., 2006). Therefore, establishing effective detection methods is essential to strengthen the epidemiological investigations of SVA and monitor its molecular evolution. Isothermal amplification techniques are currently used for SVA detection. For example, the efficiency of RT-LAMP and recombinant enzyme-mediated isothermal amplification (RT-RPA/RAA) has been demonstrated in SVA detection. The methods require only constant temperature conditions for rapid amplification and are optimal for on-site diagnosis (Li and Macdonald, 2015). However, RT-RPA/RAA is less sensitive and relatively more expensive than RT-LAMP. Consequently, in this study, RT-LAMP was selected to develop SVA assays.

In previous studies, the combination of Cas12a and Cas13a with isothermal amplification techniques has been successfully employed in nucleic acid-based assays. For instance, the DETECTR (RT-LAMP-Cas12a), SHERLOCK (RT-RPA-Cas13a), and iSCAN (RT-LAMP-Cas12a) approaches have been utilised to detect Severe Acute Respiratory Syndrome Coronavirus 2 (SARS-CoV-2) (Gootenberg et al., 2017; Chen et al., 2018; Ali et al., 2020). These CRISPR/Cas-based assays have been shown to reduce detection times, enhance sensitivity, and provide a reliable platform for the accurate identification of viruses. However, currently, no reports exist on the detection of SVA using LAMP-conjugated Cas12a or Cas13a. Consequently, this study aimed to address this knowledge gap by combining RT-LAMP and CRISPR/Cas12a to develop SVA assays, with the results evaluated using fluorescence and test-paper reporter signals. The study established that the LAMP-CRISPR/Cas12a-FQ and LAMP-CRISPR/Cas12a-FB methods are highly specific for SVA and do not cross-react with other pathogens, including FMDV, CSFV, PEDV, PCV2 or PRRSV. Subsequently, the sensitivity of the SVA detection methods was evaluated using DNA (pMD19-JC) and RNA (ST-RNA) as templates. The lowest detection limits of the DNA and RNA templates were established as 8.3 and 9.6 copies/μL, respectively. This ‘dual-template approach’ ensured the accuracy of the SAV assays, and the results were consistent with the lowest detection limits of the RT-qPCR method. The sensitivity was comparable to that of LAMP combined with the CRISPR/Cas12a method for detecting of PRRSV (Liu et al., 2021), porcine circovirus-like virus (Yu et al., 2024), SARS-CoV-2 (De Sousa et al., 2023) or African swine fever virus (Yang et al., 2022), indicating that the SVA detection methods established in this study are reliable and significant in clinical detection.

Selecting appropriate target sequences is crucial in developing diagnostic methods. The ORFs of SVA exhibit a typical L-4-3-4 structure observed in small RNA viral genomes and encode a polyprotein precursor comprising 2,180 amino acid residues (Willcocks et al., 2011). The L region encodes the leading protein Lpro: the P1 region encodes four structural proteins (VP1, VP2, VP3, and VP4), the P2 region encodes three nonstructural proteins (2A, 2B, and 2C), and the P3 region encodes four nonstructural proteins (3A, 3B, 3C, and 3D). Previous studies have demonstrated that the 5′-UTR, VP1, or 3D sequences of the SVA genome exhibit a high conservation degree, rendering them suitable for SVA detection (Hales et al., 2008). Cesar (Feronato et al., 2018) developed a nested PCR method using the VP1 gene. Bryony (Armson et al., 2019) devised two LAMP methods using the 5′-UTR and the VP1 sequence. Furthermore, Pinheiro-de-Oliveira (Pinheiro-de-Oliveira et al., 2019) developed a novel reverse transcription droplet digital PCR (RT-ddPCR) method using 3D genes as targets. Similarly, in the present study, the conserved region of the 5′-UTR was selected as the target, and three pairs of RT-LAMP primers and three crRNAs were designed in RT-LAMP and CRISPR/Cas12a detection methods for SVA.

In this study, a two-step detection process was established. First, RNA samples were extracted and subjected to RT-LAMP amplification, which can be completed at a constant temperature of 65°C for 30 min. Second, a CRISPR/Cas12a assay system was prepared. In the event that the fluorescent reporter molecule (5′-FAM-TTATT-BHQI-3′) was present within the system, the reaction was conducted at 37°C for 15 min. These results were obtained under UV light irradiation. In the event that the system contained a biotin reporter molecule (5′-FAM-TTTTTTTATTTTTTT-Biotin-3′), the test strip was detected for 5 min, following a 15-min reaction at 37°C. The results were observed with the naked eye. The entire assay was completed within 45 min. These assays are faster than methods that require hours or complex thermal cyclers, such as RT-PCR (Zhang et al., 2019), RT-qPCR (Bracht et al., 2016), nested PCR (Feronato et al., 2018), and ELISA (Yan et al., 2023). The results of our study also indicate that the CRISPR/Cas12a reaction is not contingent upon a temperature of 37°C. In contrast, a temperature range of 10–30°C is sufficient to meet the experimental requirements (Figures 3C,D). This makes the established methods more suitable for grassroots clinical testing.

The optimisation of the concentrations of both crRNA and LbCas12a, as well as ssDNA, represents a pivotal step in the CRISPR/Cas12a detection system. Conventionally, the optimisation of these concentrations in the literature has relied on a simple proportional method (Lei et al., 2022), contrasting with the approach employed in this study, which utilises an orthogonal method. This study evaluated the effects of different concentrations of LbCas12a (25–250 nM) and crRNA (25–250 nM) on the observed results through orthogonal experiments. The highest fluorescence intensity was observed at a concentration of 200 nM for LbCas12a and 100 nM for crRNA (Supplementary Figure S3). The experimental results obtained demonstrated that the data obtained through the orthogonal method were more comprehensive and reliable, thereby confirming the validity of the method. It is imperative to ascertain the requisite concentration of ssDNA for the lateral flow strip method to circumvent false positives. The minimum concentration of ssDNA is contingent on the virus under investigation and the reaction system employed. The results of this study indicate that 50 nM represents the optimal ssDNA concentration for the FB reporter gene in the RT-LAMP-Cas12a-FB assay. The high sensitivity and specificity of the established methods may be attributed to the optimisation of these conditions.

Despite the high specificity and sensitivity of the two methods developed in this study, some limitations should be acknowledged. These limitations are primarily owing to the different reaction temperatures used in the two-step method. The Bst enzyme used for RT-LAMP amplification reacts at a temperature of 60°C–65°C, whereas the Cas12a enzyme used for the CRISPR/Cas12a system reacts at a temperature of 37°C (Ding et al., 2021). The simultaneous performance of the two reactions may cause failed tests or false-positive sample results. Furthermore, opening the lid during the two-step assay procedure caused aerosol contamination (Wang et al., 2021). To ensure accurate results and prevent contamination, performing single-tube operations and maintaining a consistent reaction temperature during the process is crucial (Xu et al., 2024). Pang et al., (2020) added a CRISPR/Cas12a dry powder reagent to the lid of an RT-LAMP reagent tube and mixed it through centrifugation after RT-LAMP amplification was complete, enabling the detection of SARS-CoV-2 using the LAMP and CRISPR systems in a single tube. Researchers have also used the high-temperature resistance of the thermophilic Cas12b enzyme to achieve a single-tube reaction, alleviating the challenge of different reaction temperatures (Wang et al., 2023). Additionally, the nucleic acid extraction process of the virus to be detected constitutes an additional limiting factor for the sensitivity and economic cost of the LAMP and CRISPR systems. In this context, Ortiz-Cartagena developed an RT-LAMP-CRISPR-Cas13a method that does not require RNA extraction for detecting the SARS-CoV-2 virus in nasopharyngeal samples (Ortiz-Cartagena et al., 2022), thereby facilitating more efficient detection and reducing costs. These findings provide new insights for further optimisation of SVA detection methods.

It is noteworthy that the positive rate of samples was consistently less than 30%, despite the use of multiple testing methods established in this study. This phenomenon of low positivity rates deserves to be studied in depth and its possible causes can be tentatively attributed to the following four aspects: i) The impact of sample quality: The specification of sample collection and storage conditions may be inadequate. Considering the RNA nature of SVA, the stability of its nucleic acid is highly susceptible to changes in storage conditions. Specifically, insufficient preservation of samples at low temperatures following collection, or repeated freeze-thaw cycles during transportation, could lead to the degradation of viral RNA, ultimately resulting in a decreased detection rate. ii) Limitations of the stage of viral infection: It is well-established that some animals might be in the early stages of infection when the viral load has not yet reached the detection threshold. As such, it is advisable that, in subsequent studies, the frequency of dynamic sampling be increased to optimise the detection window period, ensuring better alignment with the onset of clinical signs (Eliya et al., 2025). iii) Selection of RNA extraction kits: It should be recognised that there can be notable differences in the efficiency of RNA extraction among RNA extraction kits from different brands. To mitigate the systematic bias arising from technical errors, it is advisable to perform batch validation of kits and conduct quantitative quality control of RNA extraction using internal reference genes. iii) Finally, the issue of pathogen cross-talk must be given due consideration. The presence of multiple pathogens with the same symptoms as SVA in clinical samples has the potential to interfere with sample collection, misclassification and test results. In summary, a comprehensive analysis of these phenomena provides potential directions for the improvement of SVA detection methods.

## 5 Conclusion

We developed two rapid, sensitive and straightforward visualisation assays using double-labelled fluorophore inactivator ssDNA (RT-LAMP-Cas12a-FQ) and fluorophore biotin ssDNA (RT-LAMP-Cas12a-FB) probes based on the RT-LAMP method and the CRISPR/Cas12a system. The methods demonstrate the capacity to detect Standard Template RNA with a detection limit as low as 9.6 copies/μL, and the entire detection process can be completed in less than 1 hour. Moreover, the methods do not require the use of sophisticated instrumentation or trained personnel, and demonstrate high specificity and satisfactory performance in the detection of clinical samples. These methods represent promising alternative for point-of-care diagnosis especially in resource-limited regions, facilitating the rapid diagnosis of vesicular diseases at the early stages of clinical manifestation in pigs, thereby enabling timely and effective disease control strategies.

## Data Availability

The original contributions presented in the study are included in the article/Supplementary Material, further inquiries can be directed to the corresponding authors.
