# Supplementary material for: Rapid molecular detection of Senecavirus A based on reverse transcription loop-mediated isothermal amplification (RT-LAMP) and CRISPR/Cas12a
Source: Front Bioeng Biotechnol. 2025 Apr 4;13:1451125. doi: 10.3389/fbioe.2025.1451125 (PMC12006090; doi:10.3389/fbioe.2025.1451125)
Supplement: Supplementary file 1 [file DataSheet1.pdf]

Supplementary Material

Rapid Molecular Detection of *Senecavirus A* Based on Reverse Transcription Loop-mediated Isothermal Amplification (RT-LAMP) and CRISPR/Cas12a

Chenghui Jiang[1, 2, †], Huibao Wang[3, †], Rongxia Guo[1, †], Rui Yang[2], Xiaoming Li[2], Ping Liu[2], Jing Wang[2], Jincai Yang[2], Yanyan Chang[1, 2, *] and Qiaoying Zeng[1, *]

[*] Correspondence: cyanyan_820116@163.com (Y.C.); zengqy@gsau.edu.cn (Q.Z.)

† These authors contributed equally to this work.

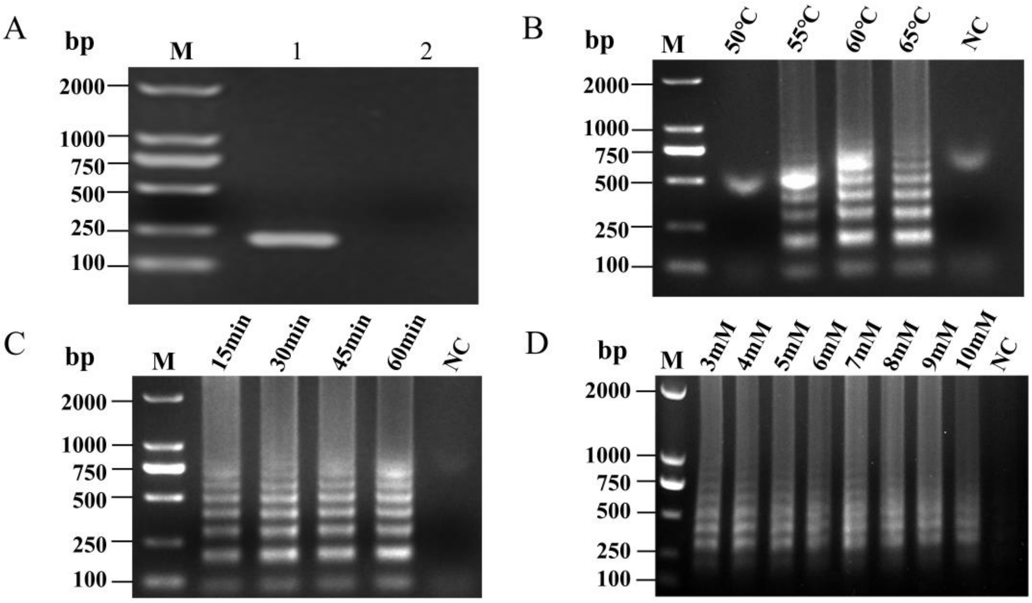

Supplementary Figure S1 Optimisation of conditions for the RT-LAMP assay. (**A**) The amplification of target genes by RT-PCR, (1) PCR product; (2) Negative control. **(B)** Optimisation of annealing temperature for RT-LAMP. **(C)** Optimisation of reaction time for RT-LAMP. **(D)** Optimisation of concentration of Mg^{2+} for RT-LAMP.

A

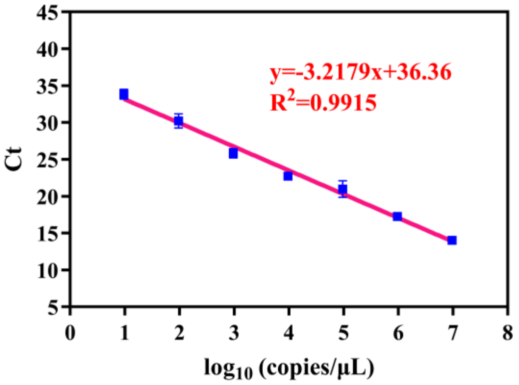

B

Sample NO.	Copies (ng/μL)	Ct (means)	Ct (SD)
**8**	9.6×10^{6}	14.83	0.57
**7**	9.6×10^{5}	17.26	0.19
**6**	9.6×10^{4}	20.98	1.32
**5**	9.6×10^{3}	22.71	0.20
**4**	9.6×10^{2}	25.82	0.16
**3**	9.6×10^{1}	30.19	0.79
**2**	9.6×10^{0}	33.83	0.76
**1**	9.6×10^{-1}	38.06	0.63
**NC**	/	/	/

Supplementary Figure S2 Standard curves and sensitivity of the RT-qPCR assay. **(A)** Standard

curves for the RT-qPCR assay (y=-3.2179x+36.36, R^2=0.9915). (**B**) Sensitivity of the RT-qPCR assay (RNA template: ST-RNA). Means indicate average, SD indicates standard deviation. In the RT-qPCR assay, samples with a cycle threshold (Ct) value of less than 35 were considered positive, while samples with a Ct value greater than 35 were considered negative.

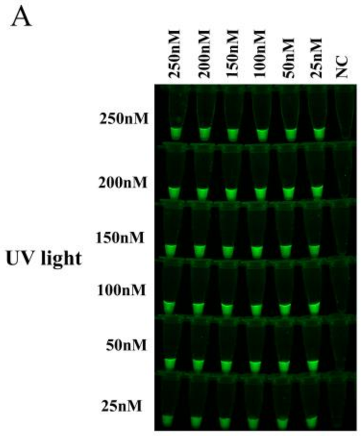

B

Cas12a protein concentration (nM)	crRNA concentration (nM)						
	250 nM	200 nM	150 nM	100 nM	50 nM	25 nM	NC
250 nM	45571	49927	48323	50975	49607	50530	5227
200 nM	54612	52657	56881	59330	54908	55368	4723
150 nM	52627	49866	48505	53832	46581	42045	4815
100 nM	47683	52375	55393	52988	53958	56427	4895
50n M	45616	50905	53995	55915	51133	51091	5381
25n M	28313	32266	31689	32160	33958	31679	4925

Supplementary Figure S3 Optimisation of the optimal concentration of crRNA and LbCas12a. (**A**) Visual results of the fluorescence of the CRISPR/Cas12a reaction at varying concentrations of crRNA and LbCas12a. (**B**) Fluorescence intensity values of the CRISPR/Cas12a reaction at varying concentrations of crRNA and LbCas12a (The values indicated by the colour red represent the maximum fluorescence intensity values).

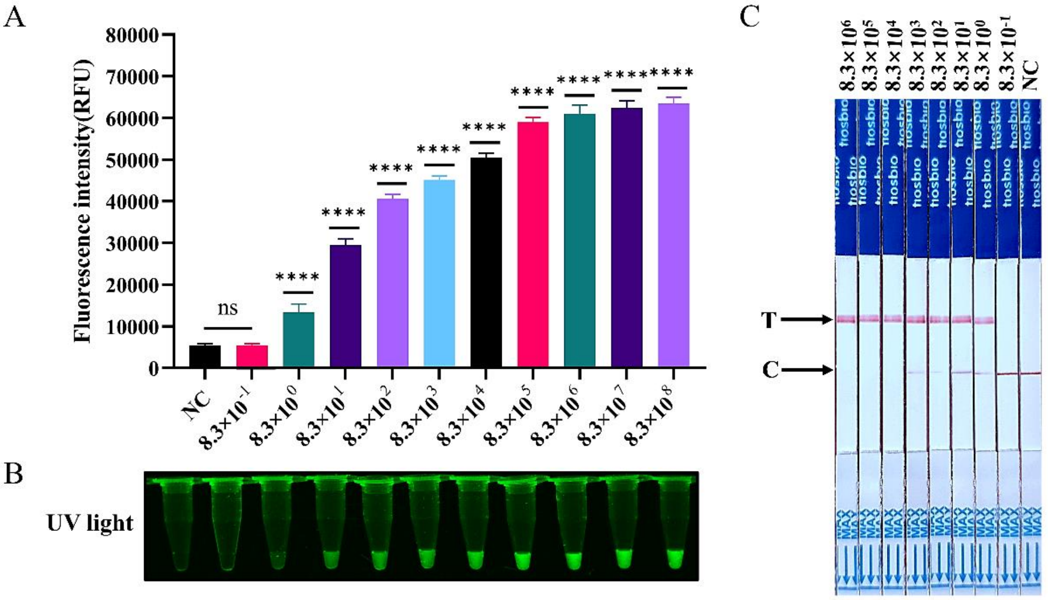

Supplementary Figure S4 Evaluation of the sensitivity of LAMP-Cas12a to detect SVA.

(A) Histogram of fluorescence intensity values of the sensitivity of the LAMP-Cas12a-FQ assay (Recombinant Plasmid (pMD19-JC) gradient ranging from 8.3×10^8 to 8.3×10^{-1} copies). **(B)** Visual fluorescence outcomes of the sensitivity of the LAMP-Cas12a-FQ assay (pMD19-JC gradient ranging from 8.3×10^8 to 8.3×10^{-1} copies). **(C)** Sensitivity of the LAMP-Cas12a-FB assay

(pMD19-JC gradient ranging from 8.3×10^6 to 8.3×10^{-1} copies).

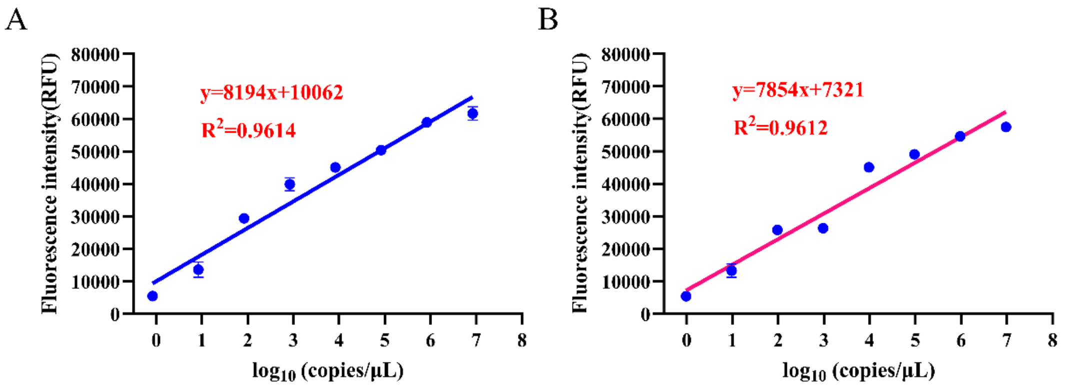

Supplementary Figure S5 The linear range of the CRISPR/Cas12a-FQ method for the detection of SVA. (**A**) The linear range of the CRISPR/Cas12a-FQ assay for SVA was assessed using pMD19-JC as a template. (**B**) The linear range of the CRISPR/Cas12a-FQ assay for SVA was assessed using Standard RNA (ST-RNA) as a template.

Supplementary Table S1 Repeatability test results of RT-LAMP-Cas12a-FQ method

Sample concentration/ (copies/μL)	CV (Coefficient of Variation) /%	
	Intra-assay	Inter-assay
9.6×10^6	3.58	7.91
9.6×10^4	3.60	8.01
9.6×10^2	4.01	8.03

Supplementary Table S2 The clinical test results and performance metric

Methods	True positive	False positive	True negative	False negative	Sensitivity (%)	Specificity (%)
RT-LAMP-Cas12a-FQ	18	0	51	0	100	100
RT-LAMP-Cas12a-FB	18	0	51	0	100	100
RT-qPCR	18	0	51	0	100	100
